# Cytotoxic and genotoxic potential of food-borne nitriles in a liver *in vitro* model

**DOI:** 10.1038/srep37631

**Published:** 2016-11-24

**Authors:** Franziska Kupke, Corinna Herz, Franziska S. Hanschen, Stefanie Platz, Grace A. Odongo, Simone Helmig, María M. Bartolomé Rodríguez, Monika Schreiner, Sascha Rohn, Evelyn Lamy

**Affiliations:** 1 University of Hamburg, Hamburg School of Food Science, Institute of Food Chemistry, Grindelallee 117, 20146 Hamburg, Germany; 2 University of Freiburg–Medical Center, Institute of Environmental Health Sciences, Molecular Preventive Medicine, Breisacher Strasse 115b, 79106 Freiburg, Germany; 3 Leibniz Institute of Vegetable and Ornamental Crops Grossbeeren/Erfurt e.V., Department of Plant Quality, Theodor-Echtermeyer-Weg 1, 14979 Grossbeeren, Germany; 4 Justus-Liebig University Giessen, Institute and Outpatient Clinic for Occupational and Social Medicine and Department of Anesthesiology, Intensive Care and Pain Medicine, Aulweg 129, 35392 Giessen, Germany; 5 Clinic for Internal Medicine II, Molecular Biology, University of Freiburg–Medical Center, Hugstetter-Straße 55, 79106 Freiburg i.Br., Germany

## Abstract

Isothiocyanates are the most intensively studied breakdown products of glucosinolates from *Brassica* plants and well recognized for their pleiotropic effects against cancer but also for their genotoxic potential. However, knowledge about the bioactivity of glucosinolate-borne nitriles in foods is very poor. As determined by GC-MS, broccoli glucosinolates mainly degrade to nitriles as breakdown products. The cytotoxicity of nitriles in human HepG2 cells and primary murine hepatocytes was marginal as compared to isothiocyanates. Toxicity of nitriles was not enhanced in CYP2E1-overexpressing HepG2 cells. In contrast, the genotoxic potential of nitriles was found to be comparable to isothiocyanates. DNA damage was persistent over a certain time period and CYP2E1-overexpression further increased the genotoxic potential of the nitriles. Based on actual *in vitro* data, no indications are given that food-borne nitriles could be relevant for cancer prevention, but could pose a certain genotoxic risk under conditions relevant for food consumption.

Results from human epidemiologic studies point towards a positive correlation between cancer prevention of many different organs and consumption of *Brassica* vegetables^1^. Yet, knowledge is still far from a complete understanding of the combinatory effects of secondary plant metabolites present in these vegetables as evident by conflicting results on the associations of *Brassica* consumption with cancer. One group of secondary plant metabolites being made responsible for the observed protective effect against cancer are glucosinolates (GSL) or more precisely their breakdown products in the form of isothiocyanates (ITC)^2^. The stability of GSL is comparatively low and they are easily hydrolyzed either by the plant-endogenous enzyme myrosinase after disruption of the plant tissue^3^, or by human microbiota-deriving myrosinase-like actvities^4^, or degraded thermally-induced (e.g. during food processing)^5^,^6^. From the GSL hydrolysis products, ITC have been studied most intensively. By now, they are well known for their pleiotropic mechanisms of interference with carcinogenesis^2^,^7^,^8^. However, enzymatic degradation not only leads to the formation of ITC, but can also lead to the formation of nitriles as main products: Especially, when plants contain modifying proteins such as the epithiospecifier protein or the nitrile-specifier proteins, enzymatic degradation of GSL can be altered in favor of nitriles^9^,^10^. Moreover, these compounds can be formed also by a non-enzymatic pathway: Several studies showed that thermally-induced degradation, e.g., during the cooking of vegetables, can result in the predominant formation of nitriles being chemically more stable than ITC^6^,^11^. Whether nitriles are also relevant nutritional factors in terms of cancer prevention by consumption of *Brassica* vegetables has been barely addressed, to date. In order to evaluate this, better knowledge on their bioactivity, but also on their concentrations in processed vegetables is indispensable. So far, some research has been done on their effect on phase II detoxification enzymes and cytotoxicity as parameters for primary and secondary/tertiary cancer prevention, respectively. A previous study compared the ability of 4-(methylsulfinyl)butyl ITC (sulforaphane) and its structural analog 5-(methylsulfinyl)pentylnitrile (4-MSOB-CN, sulforaphane nitrile) to induce the activity of mammalian phase II detoxification enzymes in Fischer 344 rats. The potential of sulforaphane nitrile to induce activity of quinone reductase was less than sulforaphane, reaching the same induction as sulforaphane in a thousand-fold concentration^12^. In contrast, administration of the nitrile crambene (3-hydroxy-4-pentenenitrile) to Fischer 344 rats [50 mg/kg BW/day] yielded an induction of hepatic quinone reductase similar to sulforaphane^13^. A recent metabolism study of 3-butenenitrile (allyl-CN) showed that the cytochrome P450 isoform CYP2E1 is involved in the allyl-CN induced lethality of mice resulting from cyanide liberation^14^. Because of their reactivity, ITC are basically able to act also as genotoxic agents, form DNA adducts, and induce DNA mutations. Some studies even report about carcinogenic effects^15^. Whether these adverse effects could also be relevant for nitriles formed from GSL is not known.

The present study aimed at initially characterizing the formation of different food-borne nitriles by enzymatic hydrolysis of GSL in selected fresh *Brassica* vegetables. In order to investigate structure dependent effects of nitriles *in vitro*, three groups (unsaturated aliphatic nitriles, aromatic nitriles, and methylthioalkylnitriles) were selected, that belong to the most common and most important groups of glucosinolate-borne nitriles. Secondly, this study evaluated the cytotoxic and genotoxic potential of food-borne nitriles in comparison to ITC using human liver HepG2 cells as well as primary murine hepatocytes. To study whether CYP2E1 can promote hepatotoxicity, CYP2E1-overexpressing HepG2 cells were used.

## Results

### Occurrence of glucosinolate-derived nitriles in *Brassica* vegetables

In order to investigate the relevance of plant-derived nitriles from fresh vegetables, the GSL content before and the nitrile content after autolysis of Brussels sprouts, broccoli, cauliflower, savoy cabbage, white cabbage, and red cabbage were studied. The results are presented in Table 1. A GC-MS chromatogram of nitriles from brokkoli is presented in the supplementary information. Individual GSL contents as well as the corresponding nitriles are given in μmol/g fresh weight (FW). Additionally, the percentage of the nitrile concentration in relation to the total content of all degradation products derived from an individual GSL are given. All *B. oleracea* vegetables formed nitriles. Especially, broccoli and cauliflower were producers of significant contents of nitriles, with 57% and 48% of all detected breakdown products being nitriles. The predominant nitriles were 4-(methylsulfinyl)butylnitrile (3-MSOP-CN) ranging from 0.01 (in cauliflower) to 0.43 μmol/g FW (in savoy cabbage), and 5-(methylsulfinyl)pentylnitrile (4-MSOB-CN) ranging from 0 μmol/g (in cauliflower) to 0.32 μmol/g FW in broccoli, that was exceptionally rich in this nitrile. Further, broccoli and Brussels sprouts had also very high nitrile ratios (65% of the degradation products of 3-MSOP GLS were 3-MSOP-CN in broccoli) with regard to total GSL except the unsaturated aliphatic ones (Table 1). However, Brussels sprouts are also a main producer of epithionitriles^16^.

### Cytotoxicity of food-borne nitriles in human liver (HepG2) cells

**Figure 1:**
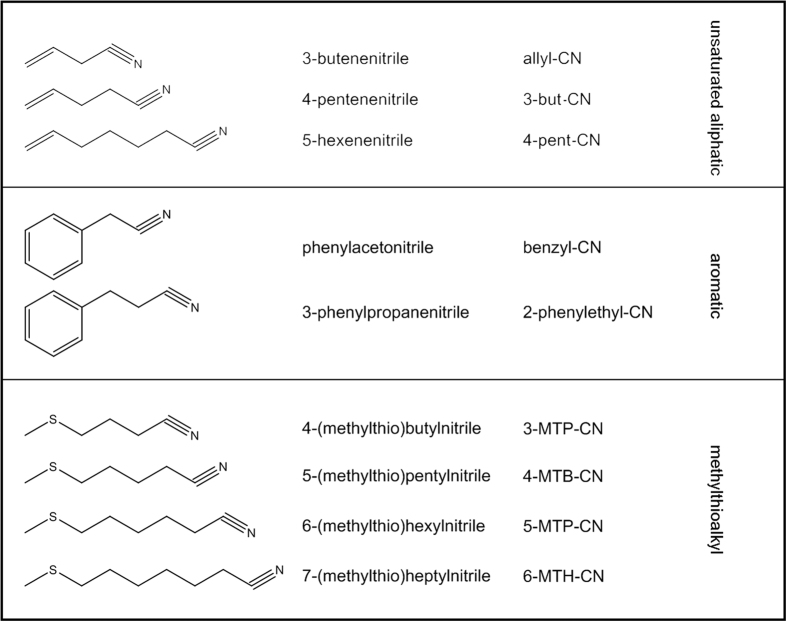
Chemical structure of the nitriles tested in this study.

**Figure 2:**
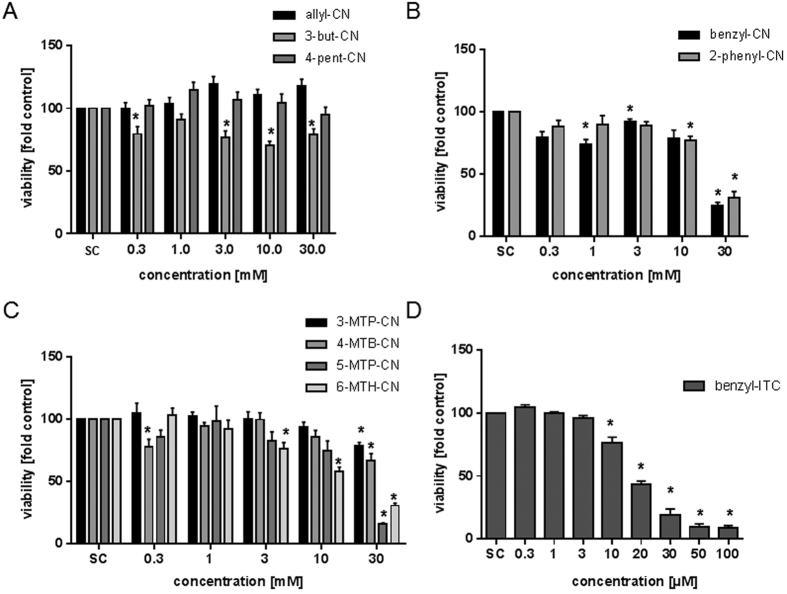
Analysis of cell viability using the WST-1 assay after a 72 h treatment with unsaturated (**A**) aromatic nitriles (**B**), methylthioalkylnitriles (**C**) and benzyl-ITC (**D**). Control: solvent control, 0–30% double distilled sterile water (**A–C**), 0.1% DMSO (**D**). Bars are mean + SEM, n = 3. *P < 0.05; asterisks indicate a significant difference between the sample and the solvent control.

As there is only limited information about secondary and tertiary cancer chemopreventive effects, cytotoxicity of unsaturated aliphatic, aromatic, and methylthioalkylnitriles (Fig. 1) against human liver cancer (HepG2) cells was studied. Keeping the toxicity of the related ITC in mind^17^,^18^, first investigations of the cytotoxicity of nitriles were carried out using concentrations ranging from 0.1 to 100 μM. However, these concentrations did not impact cell viability (data not shown). Thus, a concentration range between 0.3 to 30 mM was tested. Due to a limited solubility of nitriles in purified water, no higher concentrations than 30 mM were achieved. Treatment of HepG2 cells with unsaturated aliphatic nitriles (allyl-CN, 3-but-CN and 4-pent-CN) for 72 h did not result in significant changes in cell viability. In contrast, aromatic nitriles and methylthioalkylnitriles induced a loss of viability at concentrations exceeding 10 mM (Fig. 2). However, a significant reduction of cell viability was detected only at the highest concentration tested. The cytotoxic potential of the two aromatic nitriles was similar and lead to IC_50_ values of 19.95 mM (benzyl-CN) and 18.21 mM (2-phenylethyl-CN), respectively (Table 2). Comparative investigations with benzyl-ITC resulted in a 1000-fold stronger cytotoxic potency with an IC_50_ of 15.75 μM. Using methylthioalkylnitriles, a structure dependent increase in cytotoxicity could be observed with increasing chain length, with the lowest IC_50_ at 8.15 mM for 6-MTH-CN. The IC_50_ for 3-MTP-CN, 4-MTB-CN, and 5-MTP-CN are given in Table 2. Figure 1 shows the structural differences of these nitriles.

### Relevance of cytochrome-P450 expression for nitrile cytotoxicity

**Figure 3:**
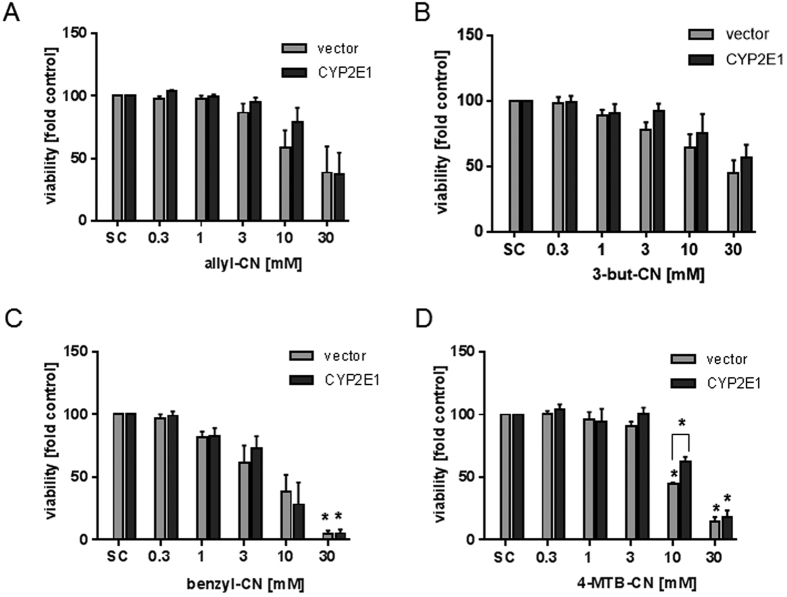
Analysis of cell viability of CYP2E1 transfected HepG2 cells and vector cells after treatment with nitriles. Cells were treated with allyl-CN (**A**), 3-but-CN (**B**), benzyl-CN (**C**) and 4-MTB-CN (**D**) for 72 hours and viability was analyzed using WST-1 assay. Bars are mean + SEM, n = 3. *P ≤ 0.05, asterisks indicate a significant difference between the sample and the solvent control: medium. CYP2E1: HepG2-CYP2E1, vector: HepG2-vector.

Recently, a study showed that the cytochrome-P450 isoform CYP2E1 is involved in the allyl-CN induced lethality of mice^14^. In contrast to normal human hepatocytes, the HepG2 cell line expresses only very low amounts of CYP2E1. This was verified by the present study using qPCR. Consequently and in order to assess whether the presence of CYP2E1 could further elevate the toxicity of the food-borne nitriles, the compounds were tested again using CYP2E1-overexpressing HepG2 cells. In comparison to wildtype HepG2 cells, the CYP2E1 expression in HepG2-CYP2E1 cells was 300 times higher. Results from two unsaturated aliphatic nitriles (allyl-CN, 3-but-CN), the aromatic nitrile benzyl-CN, and the methylthioalkylnitrile 4-MTB-CN are shown in Fig. 3. Only 10 mM 4-MTB-CN showed a significant difference in cytotoxicity between CYP2E1-overexpressing and vector control cells. To the best of our knowledge, there are no other proteins described in the literature that have an influence on the cytotoxicity of nitriles other than CYP2E1.

### Cytotoxicity of food-borne nitriles in normal murine hepatocytes

**Figure 4:**
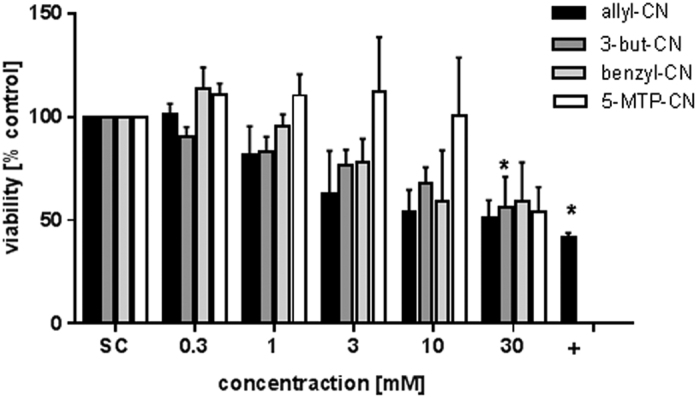
Analysis of cell viability in primary murine hepatocytes after a 72 hours treatment with nitriles using WST-1 assay. +: Positive control, 0.05% triton-X. Bars are mean + SEM, n ≥ 3. *P < 0.05; asterisks indicate a significant difference between the sample and the solvent control, medium.

To analyze the effect of nitriles on primary hepatocytes, allyl-CN, 3-butyl-CN, benzyl-CN, and 4-MTB-CN were studied in hepatocytes freshly isolated from mice. Allyl-CN, 3-but-CN and benzyl-CN significantly decreased the viability of hepatocytes in a concentration-dependent manner (Fig. 4) as analyzed by WST-1 assay after 72 h of exposure. This loss in cell viability was higher for allyl-CN and 3-but-CN than in HepG2 cells (Fig. 2A). For 4-MTB-CN, a viability loss was detected only at 30 mM, comparable with the effect in HepG2 cells (Fig. 2C).

### Genotoxicity of food-borne nitriles in HepG2 cells in comparison to isothiocyanates and the relevance of CYP2E1

**Figure 5:**
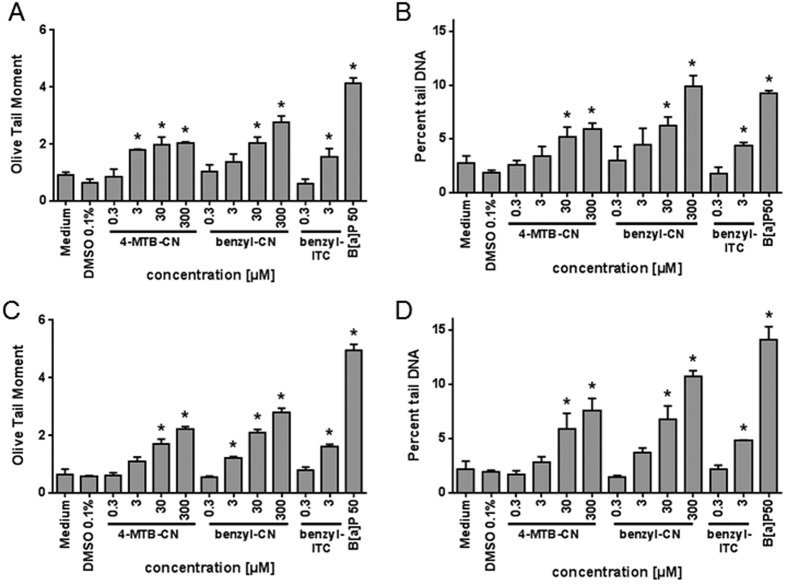
Analysis of DNA strand breaks of HepG2 cells after treatment with 4-MTB-CN, benzyl-CN and benzyl-ITC for 24 hours (**A,B**) or after 24 hours repair (**C,D**) using Comet assay. Data are presented as mean + SEM, B[α]P: Benzo[*a*]pyrene, positive control, n = 3, *P ≤ 0.05 asterisks indicate a significant difference between the sample and the solvent control: medium for nitriles or 0.1% DMSO for benzyl-ITC and B[α]P.

**Figure 6:**
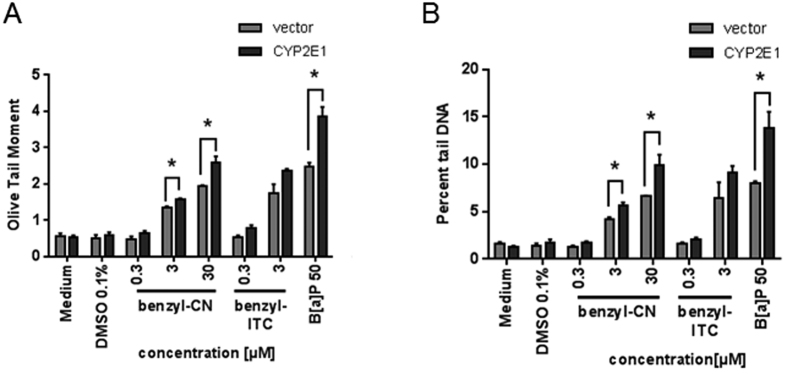
Analysis of DNA strand breaks of CYP2E1 transfected HepG2 and vector cells, treated with benzyl-CN and benzyl-ITC for 24 hours using the Comet assay. Data are presented as mean + SEM. to corresponding control, for nitriles medium (solvent), for benzyl-ITC and B[α]P 0.1% DMSO (solvent), B[α]P 50 μM: Benzo[*a*]pyrene, positive control, n = 3, *P ≤ 0.05 significance is related to the solvent control.

Risk-benefit evaluation of bioactive compounds formed in *Brassica* vegetables is not only based on its cancer preventive potency, but also on further aspects such as genotoxicity^15^. ITC are comparatively electrophilic compounds that have been shown to act genotoxic, at least *in vitro*^19^,^20^. Representatively, the genotoxicity was investigated in terms of DNA break induction for 4-MTB-CN and benzyl-CN in comparison to benzyl-ITC using the *Comet assay* (Fig. 5). 4-MTB-CN and benzyl-CN induced a concentration-dependent increase in DNA strand breaks as determined by the Olive Tail Moment and percent tail DNA (Fig. 5A and B). A significant increase in DNA strand breaks was evident for 4-MTB-CN and benzyl-CN at a concentration of ≥3 μM. The DNA damage induced by 3 μM benzyl-CN was then comparable with the effect of benzyl ITC at the same concentration (Fig. 5A and B). After 24 h of recovery, the DNA damage still persisted (Fig. 5C and D). Analog to the cytotoxicity tests, next the effect of CYP2E1 expression on the genotoxic potency of nitriles was analyzed (Fig. 6). CYP2E1-overexpression caused a significant increment of DNA strand breaks at concentrations of ≥3 μM benzyl-CN as compared to vector control cells. This indicates a relevance of CYP2E1 for the genotoxic potential of this nitrile.

## Discussion

Many *Brassica* vegetables contain alkenyl-GSL that can be hydrolyzed to ITCs, nitriles, and epithionitriles. However, studies on the occurrence of food-borne nitriles and the knowledge about their biological effects are still not comprehensively. In the present study, the predominant nitriles in vegetables were 3-MSOP-CN and 4-MSOB-CN, the latter ranging from 0 μmol/g (in cauliflower) to 0.32 μmol/g FW in broccoli that was exceptionally rich in this nitrile. This is in line with data reported in the literature. In the study of Matusheski *et al*. (2004), 4-MSOB-CN concentrations ranged from 0.35 to 1.50 μmol/g fresh weight (in broccoli). Moreover, the preferential formation of 3-MSOB-CN and 4-MSOB-CN compared to the corresponding ITC has been already observed by Daxenbichler *et al*.^21^ for freshly autolyzed cabbage.

In the actual study, Brussels sprouts had also very high nitrile ratios with regard to total GSL, except the unsaturated aliphatic ones. The latter released, probably due to a high ESP activity, the corresponding epithionitriles and therefore Brussels sprouts were in total a producer of epithionitriles which is in agreement to others^21^. However, the highest total nitrile concentration with 0.58 μmol/g FW was found in savoy cabbage, although it was, likewise to red cabbage, also a well-known producer of ITC. This was due to a high content of 3-MSOP-GSL and its hydrolysis products comprised to 37% of the nitrile. Therefore, consumption of 200 g savoy cabbage would mean an ingestion of 86 μmol of this nitrile. Moreover, it has been shown that the aliphatic nitrile allyl-CN resulting from allyl-GSL occurred, in all of the *B. oleracea* vegetables investigated, except in broccoli. Consistently, in all analyzed *B. oleracea* vegetables also 3-MTP-CN was formed. The occurrence of this nitrile has been already reported for cabbage and cauliflower^21^,^22^. However, during the last decades, ITC were made responsible for any physiological effects of *Brassica* consumption, e.g., also for cancer prevention. Therefore, it is mandatorily necessary to also clarify possible effects of food-borne nitriles.

As shown by Matusheski and Jeffery^12^, the ability of the sulforaphane nitrile to induce quinone reductase was about 1000-fold less than that of sulforaphane itself. Sulforaphane induced quinone reductase at a concentration of 2.5 μM, reaching a maximum induction of 3.1-fold, whereas the corresponding nitrile reached a similar induction only at a concentration of 2 mM^12^. In a recently published study, strong interference of benzyl-ITC at ≥3 μM with the cellular pro-inflammatory response including prostaglandin PGE2 release was shown in activated human T lymphocytes. In contrast, benzyl-CN did not influence PGE2 release even at a concentration of 1 mM^23^. These results confirm the observation that nitriles affect their target less than their corresponding ITC when applied at similar concentrations. In the present study, the cytotoxic potential of two aromatic nitriles (benzyl-CN and 2-phenylethyl-CN) was comparable. Investigations with benzyl-ITC resulted in a 1000-fold stronger cytotoxic potency compared to the nitriles (Fig. 2D). Lui *et al*. (2003) determined the IC_50_ of benzyl-ITC using the MTT-assay in the head and neck squamous carcinoma cell line UM-2BB with a value of 17 μM. These data imply that in comparison to ITC, food-borne nitriles are far less toxic to human cancer cells *in vitro*. Indications are given that a minor structural difference such as the loss of a methyl group, could lead to different structurally-dependent effects being involved in cytotoxicity mechanism. Ahmed & Farooqui^24^ reported for rats that the toxicity of aliphatic nitriles not only diversifies with cyanide liberation but is also highly dependent on their chemical structure. In that study, saturated nitriles at amounts of 0.7 mmol produced symptoms of poisoning similar to the symptoms produced by inorganic cyanide (0.15 mmol). In contrast, unsaturated nitriles with amounts of 1.7 mmol showed only moderate symptoms^24^. In the present study, methylthioalkylnitriles with increasing chain length lead to a structure-dependent increase in cytotoxicity, with 6-MTH-CN being the most toxic substance (Table 2). It has to be taken into account that concentrations inducing cell toxicity were still very high. In a previous study, the IC_50_ of 4-MTB-ITC was determined using the neutral red retention assay with 23.18 μM in HepG2 cells^18^. This ITC, again, is a 1000-fold more potent in inducing cell death as compared to the cytotoxicity observed with the nitrile 4-MTB-CN in the present study. In accordance to others^24^, data of the present study also imply that no relevant cytotoxicity occurs when an aliphatic moiety in the nitrile is present. However, presence of sulfur or an aromatic ring in the chemical structure of the chain led to an increased cytotoxicity in HepG2 cells. This effect was still very weak as compared to ITC, underlining the hypotheses already described in the literature^12^,^13^. Keck *et al*.^13^ reported that treatment of mouse hepatoma cells (Hepa1c1c7) with the brassicacaeous nitrile crambene (2-OH-3-but-CN) in a concentration of 5 mM for 72 h, diminished cell proliferation by 76%. Of those, cell viability was reduced by 12%. Thus, tested concentrations in this study are comparatively high and not expected to occur *in vivo* after consumption of processed or non-processed *Brassica* foods.

Nitriles seem only to be activated by CYP2E1 and other CYPs. Thus, it was suggested that the toxic effects result from cyanide liberation following the activation of certain enzymes^14^. Although cytotoxicity of nitriles was not affected by CYP2E1-overexpression in HepG2 cells in the present study, this was the case for genotoxicity. Here, a genotoxic effect was already significant at a concentration of ≥3 μM. Moreover, CYP2E1 overexpression increased genotoxicity. Whereas ITC are well known genotoxic agents *in vitro*, this was not known for nitriles. For example Lamy *et al*. (2009) investigated the genotoxic potential of 3-MTP-ITC, 4-MTB-ITC, and 5-(methylthio)pentyl ITC (5-MTP-ITC) in HepG2 cells using the *Comet assay* as well as the micronucleus test. That study concluded that the ITC induced DNA damage and mutagenicity at concentrations exceeding 1 μM. Based on the present data nitriles seem to be as potent as ITC to induce DNA damage and this damage seems to be persistent. The intensity of DNA strand breaks of 3 μM benzyl-CN was comparable with the effect of benzyl-ITC in the same concentration. *In vitro* experiments with benzyl-ITC showed characteristic concentration-dependent genotoxic effects under different conditions (dependent on the cell line or endpoint)^25^. Comparatively weaker effects were observed in *in vivo* experiments. The genotoxicity of benzyl-ITC was reduced by bovine serum albumin and human body fluids, which suggests that benzyl-ITC is inactivated under *in vivo* conditions^25^. If nitriles are also detoxified *in vivo*, needs to be clarified in future studies.

Under the conditions applied in the present study, *B. oleracea* vegetables released high amounts of nitriles upon hydrolysis of GSL. Especially broccoli and cauliflower were intense producers of nitriles. The highest total nitrile concentration was detected in savoy cabbage, though comprising mainly of 3-MSOP-CN. With regard to cell culture experiments, tested nitriles were cytotoxic towards cancer cells only when being applied in 1000-fold higher concentrations, compared to the ITC. Thus, an impact of nitriles for a secondary and tertiary cancer preventive potential by *Brassica* vegetables seems neglectable. Moreover, with regard to common habits of food intake, an indication for a preventive potential of GSL-derived nitriles can still not be given with the present data. However, the tested nitriles showed genotoxic potential in HepG2 cells, comparable in effect to their corresponding ITC. Thus, the preference of nitrile instead of ITC formation under certain food processing conditions should be taken into account. Moreover, the weak cytotoxic activity of nitriles while being quite potent genotoxic agents should be considered carefully, as well. This genotoxic activity of nitriles, as shown in this study, can be considered as a risk factor in cancer prevention. Thus, this could be one relevant factor being involved in the contradictory observations made with cancer prevention studies using *Brassica* vegetables. As only three structure-types of nitriles were tested in the present study, further research with nitriles differing in their chemical structure is necessary. Based on the *in vitro* data presented, there are no indications that nitriles affect health without a preceding activation by CYP enzymes. However, with respect to genotoxicity data, it cannot be ruled out that nitriles pose a risk under conditions relevant for food consumption.

## Materials and Methods

### Chemicals

Benzonitrile (≥99.9%), 3-phenylpropanenitrile (2-phenylethyl-CN, ≥99%), 3-butenenitrile (allyl-CN, ≥98%), 4-pentenenitrile (3-but-CN, ≥97%), 2-propenyl GSL hydrate (sinigrin, allyl-GSL, ≥99%), 2-propenyl ITC (allyl-ITC, ≥99%), 3-(methylthio)propyl ITC (3-MTP-ITC, ≥98%), 4-bromobutyronitrile (≥97%), 6-bromohexanenitrile (≥95%), 7-bromoheptanenitrile (≥90%), triton X-100 (laboratory grade), and trypan blue powder were purchased from Sigma-Aldrich GmbH (Steinheim, Germany); 2-phenylethyl ITC (2-phenylethyl-ITC; ≥99%) was purchased from SAFC Supply Solutions (St. Louis, Missouri, USA); benzyl ITC (benzyl-ITC, ≥97%) and phenylacetonitrile (benzyl-CN, ≥98%) from Lancaster Synthesis Ltd. (Morecambe, UK); 3-indoleacetonitrile (IAN, ≥98%) from Acros Organics (Fischer Scientific GmbH, Schwerte, Germany); 5-hexenenitrile (4-pent-CN, ≥95%), 3-butenyl ITC (3-but-ITC, ≥95%), 4-pentenyl ITC (4-pent-ITC, ≥95%) and 5-bromovaleronitrile (≥95%) were purchased from TCI Deutschland GmbH (Eschborn, Germany); 3-(methylsulfinyl)propyl ITC (3-MSOP-ITC, ≥97%) and 4-(methylthio)butyl ITC (4-MTB-ITC, ≥98%) were purchased from Santa Cruz Biotechnology (Heidelberg, Germany); 4-(methylsulfinyl)butyl ITC (4-MSOB-ITC, ≥98%) was purchased from Enzo Life Sciences GmbH (Lörrach, Germany); 1-cyano-2,3-epithiopropane (CETP, ≥95%) was purchased from Taros Chemicals GmbH Co. KG (Dortmund, Germany); methanol (>99.9) and Na_2_SO_4_ were purchased from VWR International GmbH (Darmstadt, Germany); methylene chloride (GC Ultra Grade) was purchased from Carl Roth GmbH (Karlsruhe, Germany); diethyl ether (≥99%) was purchased from BCD Chemie GmbH (Hamburg, Germany). 4-(methylthio)butylnitrile (3-MTP-CN), 5-(methylthio)pentylnitrile (4-MTB-CN), 6-(methylthio)hexylnitrile (5-MTP-CN) and 7-(methylthio)heptylnitrile (6-MTH-CN) were synthesized as described in the section Chemical synthesis; trypsine, trypsine/EDTA, low glucose Dulbecco’s modified eagle medium, fetal calf serum and L-glutamine (200 mM) were purchased from PAA Laboratories GmbH (Pasching, Austria) and Gibco^®^, Life Technologies GmbH (Darmstadt, Germany). Penicillin-Streptomycin solution was purchased from Life Technologies GmbH (Darmstadt, Germany). Acetonitrile (Ultra Gradient HPLC grade) was purchased from J.T. Baker (Deventer, Nederland). RNeasy mini Isolation kit and RNase-free DNase kit were purchased from QIAGEN GmbH (Hilden, Germany). Absolut^TM^ QPCR SYBR Green Capillary Mixes was purchased from Fisher Scientific (Pittsburgh, PA, USA).

### Chemical synthesis of methylthioalkylnitriles

Due to the poor commercial availability of methylthioalkylnitriles, the four tested components have been synthesized according to Moon *et al*.^26^: 4-(methylthio)butylnitrile (3-MTP-CN). Sodium thiomethoxide (0.23 g, 3.3 mmol; 95%, Sigma-Aldrich GmbH, Steinheim, Germany) was dissolved in 5 mL methanol and gradually added to a solution of 4-bromobutyronitrile (0.5 g, 3.4 mmol; 97%) in 5 mL methanol. The mixture reacted 7 h under reflux conditions. Subsequent to evaporating the methanol *in vacuo*, the resulting product was dissolved in 13 mL water and extracted with diethyl ether (3 × 25 mL). The combined organic extracts were dried over sodium sulfate and the solvent was removed. 5-(methylthio)pentylnitrile (4-MTB-CN), 6-(methylthio)hexylnitrile (5-MTP-CN), 7-(methylthio)heptylnitrile (6-MTH-CN). The nitriles were synthesized according to the same method as described above. 0.5 g 5-bromovaleronitrile (3.1 mmol) to synthesize 5-(methylthio)pentylnitrile, 0.6 g 6-bromohexanenitrile (3.4 mmol) for 6-(methylthio)hexylnitrile, and 0.6 g 7-bromoheptanenitrile (3.2 mmol) for synthesis of 7-(methylthio)heptylnitrile were used. The amount of sodium thiomethoxide was kept unaltered: 0.23 g (3.3 mmol).

### Plant Material and Sample Preparation

For determining the occurrence of nitriles in vegetables, selected *Brassica* vegetables were grown at the experimental sites of the Leibniz Institute of Vegetable and Ornamental Crops Grossbeeren/Erfurt e.V., Grossbeeren, Germany and harvested during autumn 2013. Fertilization, irrigation, and plant protection corresponded to standard cultivation procedures of *Brassica* vegetables. As economically important horticultural *Brassica* species, white cabbage *(Brassica oleracea* var. *capitata* f. *alba*, cv. Reaktion), red cabbage (*Brassica oleracea* var. *capitata* f. *rubra*, cv. Pesaro), savoy cabbage (*Brassica oleracea* var. *sabauda*, cv. Traviata), cauliflower (*Brassica oleracea* var. *botrytis*, cv. Chambord), broccoli (*Brassica oleracea* var. *italica,* cv. *Iron Man*), and Brussels sprouts (*Brassica oleracea* var. *gemmifera*, cv. Crispus) were chosen. For determining the amount of the different compounds, fully developed heads (cabbages, broccoli, and cauliflower) or fully developed leaf buds (Brussels sprouts) were harvested. For each *Brassica* vegetable, five biological replicates were taken using a representative aliquot for the determination of GSL and nitriles. Vegetables were free of injuries or pests and analyzed without delay after harvest. *Brassica* head forming and inflorescence vegetables were cut into 5 × 5 cm pieces (cabbages, broccoli, cauliflower), the leaf buds of Brussels sprouts were cut in half and mixed. Half of each sample was immediately frozen at −50 °C and lyophilized for GSL determination. From the other half, an aliquot was crushed with a homogenizer (Edmund Bühler H04, Tübingen, Germany) after addition of an aliquot of water (one part of the plant/one part of water). From the homogenized sample an aliquot (1 g containing 500 mg of water) was immediately subjected to the analysis of nitriles.

### Cell culture

HepG2 cells were purchased from the German Collection of Microorganisms and Cell Cultures (DSMZ, Braunschweig, Germany). HepG2 cells which constitutively express human CYP2E1 (E47 cells, HepG2-CYP2E1) and control HepG2 cells transfected with empty vector (C34 cells, HepG2- vector), originally established by Wu and Cederbaum^27^, were kindly provided by C. Hellerbrand (University Medical Center Regensburg, Germany). The cells were cultured in low glucose Dulbecco’s modified eagle medium supplemented with 15% fetal calf serum and 1% Pen/Strep in an atmosphere with 5% CO_2_ at 37 °C and 95% air humidity.

### Determination of the drug effect in primary murine hepatocytes

Murine hepatocytes were obtained from 8–16 week-old, male C57BL6 mice (Charles River Laboratory, Sulzfeld, Germany) as described elsewhere^18^. The institutional and national guidelines for the care and use of animals were followed, and all experimental procedures were approved by the institutional Animal Care and Use Committee at the University of Freiburg according to § 8.I of the Animal Welfare Act. 2–2.2 × 105 cells were plated in 24 well plates. The cells were left undisturbed for 4 h in a 5% CO_2_ atmosphere at 37 °C and then the medium was changed to serum free Williams’ Medium E supplemented with 100 nM dexamethasone. Within 24 h the cells were exposed to the test compounds and controls for 72 h.

### Determination of glucosinolates from *Brassica* vegetables

GSL concentration in vegetable samples was determined as desulfo-GSL using the DIN EN ISO 9167–1 based method as previously described in ref. 28. Briefly 15 mg of lyophilized plant material were extracted methanolically, GLS were desulfated and analyzed by UHPLC-DAD using a Poroshell 120 EC-C18 column, 100 mm × 2.1, particle size 2.7 μm (Agilent Technologies and a gradient comprising of water and acetonitrile as reported previously^28^. Desulfo-GSL were identified by comparing retention times and UV absorption spectra with those of authentic standards. Quantification was done by using an external calibration curve with allyl-GSL and a wavelength of 229 nm.

### Determination of nitriles in *Brassica* vegetables

For the determination of nitriles, the method reported previously by Witzel *et al*. (2013) was adapted. An aliquot of vegetable sample (1 g consisting of 500 mg plant material and 500 mg of water) was weighted into a centrifugal tube, benzonitrile as internal standard (IST) was added, analytes were extracted with methylene chloride, and analyzed by GC-MS as described previously^29^ using a SGE BP5MS column 30 m × 0.25 mm × 0.25 μm (VWR International GmbH, Darmstadt, Germany). Analytes were identified by comparing mass spectra and retention times with those of authentic standards and with literature data^30^. Mass spectral data of selected *Brassica* nitriles are presented in the supplementary information. Analyte content was calculated using benzonitrile as internal standard and corresponding response factors (RF): CETP (RF = 2.10), allyl-ITC (RF = 1.70), allyl-CN (RF = 3.70), 3-but-ITC (RF = 1.06), 3-but-CN (RF = 2.45), 3-MTP-ITC RF = 1.07, 4-MTB-ITC (RF = 0.76), 3-MSOP-ITC (RF = 1.74), 4-MSOB-ITC (RF = 1.80), benzyl-ITC (RF = 0.72), benzyl-CN (RF = 0.72), 2-phenylethyl-ITC (RF = 0.68), 2-phenylethyl-CN (RF = 0.82), IAN (RF = 0.84); all relative to benzonitrile. For those compounds not commercially available, the RF of the chemically most similar compound was used: Other epithionitriles than CETP were calculated at hand of the RF of CETP. 5-Vinyl-1,3-oxazolidine-2-thione (OZT) was calculated with the RF of 3-but-ITC, 2-OH-3-but-CN was calculated with the RF of 3-but-CN. The corresponding nitriles of 3-(methylthio)propyl (3-MTP) GSL, 4-(methylthio)butyl (4-MTB) GSL, 3-(methylsulfinyl)propyl (3-MSOP), and 4-(methylsulfinyl)butyl (4-MSOB) GSL were calculated with the RF of the corresponding ITC. 4-Methoxy-3-indoleacetonitrile (4-methoxy-IAN) was quantified with the RF of IAN. All solvents were of HPLC or GC-MS grade. Water was of Milli-Q quality.

### Determination of cytotoxicity

Cytotoxicity was determined with the WST-1 assay (Roche Diagnostics Deutschland GmbH, Mannheim, Germany). The assay is based on the ability of viable cells to cleave the tetrazolium salt WST-1 to formazan by their mitochondrial succinate-tetrazolium-reductase system. Cells were cultured at an initial density of 5000 cells/well in 96-well plates for 48 h. Then, cells were treated with the nitriles (0.3 to 30 mM, solved in distilled water) for 72 h. As positive control, 0.01% Triton X-100 was used. Following compound exposure and incubation, the WST 1 assay was performed according to the manufacturer’s instructions.

### Single cell gel electrophoresis assay (*Comet Assay*)

The single cell gel electrophoresis assay also known as *Comet assay* was carried out as described earlier with slight modifications^20^. The percent tail DNA and the Olive Tail Moment (OTM) were calculated as indicators of DNA damage. For each sample, 102 systematically screened cells were evaluated. Briefly, HepG2 cells were seeded onto well plates. After 48 h of growth, the cells were exposed to the nitriles or ITC for 24 h. As positive control 50 μM B(α)P and as solvent control distilled water or 0.1% DMSO was used. To analyze the repair capacity of HepG2 cells, the test substances were removed after 24 h and incubated for another 24 h before the assay was terminated.

### Real-time polymerase chain reaction

Total RNA was isolated with the RNeasy mini Isolation kit from QIAGEN GmbH followed by a purification step using the RNase-free DNase kit from QIAGEN GmbH according to manufacturer’s instructions. 5 μg RNA of each cell line was dried by a vacuum concentrator (Eppendorf AG, Hamburg, Germany). Isolated RNA was re-suspended in 10 μL of RNAse-free water. Each sample was treated twice with 2 μL RNAse-free DNAse 1 unit/μL (QIAGEN GmbH) for 10 min at 37 °C to eliminate remaining DNA. The RNA prepared was reverse-transcribed as described elsewhere^31^.

For quantitative comparison of CYP2E1 mRNA levels real-time polymerase chain reaction (qPCR) was performed using SYBR-green fluorescence in a LightCycler^®^ System (Roche Diagnostic GmbH, Mannheim, Germany). After optimization of the PCR conditions, amplification efficiency was tested in standard curves using serial cDNA dilutions. The correlation coefficient had to be above 0.9 and the slope around −3.5. Amplification specificity was checked using melting curves. Gene expression was related to the mean expression of the four housekeeping genes (HSK) beta-2-microglobulin (B2M), beta-actin (β-Actin), hypoxanthine-guanine phosphoribosyltransferase (HPRT) and 5-monooxygenase activation protein, zeta (YWAHZ). Calculations of expression was performed with the 2^−ΔΔCT^ method according to Pfaffl *et al*.^32^. The sequence of the specific primers were CYP2E1_for_ (5′CCT ACA TGG ATG CTG TGG TG-3), CYP2E1_rev_ (5′TGG GGA TGA GGT ATC CTC TG-3′), B2M_for_ (5′-ACTGAATTCACCCCCACTGA-3′), B2M_rev_ (5′-CCTCCATGATGATGCTTACA-3′), β-Actin _for_ (5′-CTG GAA CGG TGA AGG TGA CA-3′), β-Actin_rev_ (5′-AAG GGA CTT CCT GTA ACA ACG CA-3′) HPRT_for_ (5′-ATG CTG AGG ATT TGG AAA GGG-3′), HPRT_rev_ (5′GCA CAC AGA GGG CTA CAA TG-3′) and YWAHZ_for_ (5′ACT TTT GGT ACA TTG TGG CTT CAA-3′) YWAHZ_rev_ (5′CCG CCA GGA CAA ACC AGT AT -3′)^33^,^34^,^35^,^36^. PCR reactions were carried out in a final volume of 20 μL using 1x Absolut^TM^ QPCR SYBR Green Capillary Mixes, 300 nM of HSK primers or 600 nM of CYP2E1 primers and 2 μL cDNA. The PCR conditions for CYP2E1 were as follows: Initial denaturation 15 min at 95 °C, touch down PCR 2 cycles of 95 °C for 10 s, 65 °C for 10 s, 72 °C for 25 s; 2 cycles of 95 °C for 10 s, 63 °C for 10 s, 72 °C for 25 s; 2 cycles of 95 °C for 10 s, 61 °C for 10 s, 72 °C for 25 s; 45 cycles of 95 °C for 10 s, 59 °C for 10 s, 72 °C for 25 s. The PCR conditions for HSK were as follows: Initial denaturation 15 min at 95 °C, PCR 45 cycles of 95 °C for 15 s, 63 °C (B2M), 61 °C (HPRT), 56 °C (β-Actin), 58 °C (YWHAZ) for 15 s, 72 °C for 15 s. All measurements were made without information about the origin of the samples and were performed in duplicate.

### Statistical Analysis

The median growth inhibitory concentration (IC_50_) for compound cytotoxicity was calculated using Graphpad Prism 5.0 software (La Jolla, CA). After logarithmic transformation, IC_50_ values were calculated with equation 1:


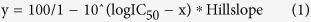


All experiments were performed using four replicates and were repeated at least two times, independently. Significance of difference between the treatment groups in the *Comet assays* was analyzed using the ordinary one-way ANOVA^37^ and WST-1 data was analyzed by using the one way ANOVA followed by Bonferroni correction. The comparison of the two groups was analyzed using an unpaired t-test. The differences for both tests with p ≤ 0.05 (*) were considered statistically significant.

## Additional Information

**How to cite this article**: Kupke, F. *et al*. Cytotoxic and genotoxic potential of food-borne nitriles in a liver *in vitro* model. *Sci. Rep.* **6**, 37631; doi: 10.1038/srep37631 (2016).

**Publisher’s note:** Springer Nature remains neutral with regard to jurisdictional claims in published maps and institutional affiliations.

## Supplementary Material

Supplementary Information

## Figures and Tables

**Table 1:** Glucosinolate (GSL) content and enzymatically-formed nitriles in *Brassica* vegetables in μmol/g FW.

GSL	nitrile (μmol/g FW)	Brussels sprouts	broccoli	cauliflower	savoy cabbage	white cabbage	red cabbage
allyl-GSL		0.646 ± 0.046	0.004 ± 0.001	0.095 ± 0.020	1.292 ± 0.301	0.582 ± 0.193	0.360 ± 0.018
	allyl-CN	0.028 ± 0.008 (3%)	n.d.	0.015 ± 0.011 (12%)	0.094 ± 0.026 (9%)	0.059 ± 0.024 (11%)	0.010 ± 0.003 (3%)
3-but-GSL		0.095 ± 0.010	n.d.	n.d.	0.022 ± 0.006	0.023 ± 0.010	0.224 ± 0.025
	3-but-CN	0.008 ± 0.002 (7%)	n.d.	n.d.	n.d.	n.d.	n.d.
2-OH-3-but-GSL		0.243 ± 0.041	n.d.	0.002 ± 0.005	0.069 ± 0.027	0.043 ± 0.015	0.458 ± 0.046
	2-OH-3-but-CN	n.d.	n.d.	n.d.	n.d.	n.d.	0.036 ± 0.007 (10%)
3-MTP-GSL		0.012 ± 0.001	n.d.	0.050 ± 0.019	0.027 ± 0.008	0.012 ± 0.008	0.010 ± 0.003
	3-MTP-CN	0.007 ± 0.004 (100%)	0.004 ± 0.002 (100%)	0.10 ± 0.057 (91%)	0.019 ± 0.008 (72%)	0.006 ± 0.002 (30%)	0.003 ± 0.001 (20%)
4-MTB-GSL		n.d.	0.013 ± 0.013	n.d.	n.d.	0.007 ± 0.010	0.029 ± 0.008
	4-MTB-CN	n.d.	0.005 ± 0.001 (47%)	0.003 ± 0.001 (100%)	n.d.	n.d.	0.005 ± 0.001 (10%)
3-MSOP-GSL		0.707 ± 0.066	0.121 ± 0.013	0.160 ± 0.035	1.641 ± 0.295	0.609 ± 0.091	0.471 ± 0.061
	3-MSOP-CN	0.188 ± 0.052 (89%)	0.104 ± 0.154 (65%)	0.011 ± 0.017 (41%)	0.433 ± 0.089 (30%)	0.177 ± 0.045 (49%)	0.108 ± 0.012 (25%)
4-MSOB-GSL		0.121 ± 0.019	0.690 ± 0.132	n.d.	0.096 ± 0.020	0.064 ± 0.023	1.003 ± 0.204
	4-MSOB-CN	0.040 ± 0.017 (93%)	0.322 ± 0.078 (54%)	n.d.	0.031 ± 0.043 (49%)	0.009 ± 0.012 (54%)	0.256 ± 0.037 (22%)
benzyl-GSL		0.025 ± 0.006	n.d.	n.d.	n.d.	n.d.	n.d.
	benzyl-CN	0.030 ± 0.004 (90%)	n.d.	n.d.	n.d.	n.d.	n.d.
2-Phenylethyl-GSL		n.d.	n.d.	n.d.	0.033 ± 0.005	n.d.	n.d.
	2-Phenylethyl-CN	0.007 ± 0.003 (100%)	0.001 ± 0.001 (100%)	n.d.	0.006 ± 0.004 (41%)	n.d.	n.d.
4-OH-I3M-GSL		0.061 ± 0.013	0.035 ± 0.002	0.026 ± 0.009	0.012 ± 0.006	0.016 ± 0.007	0.049 ± 0.003
I3M-GSL		0.455 ± 0.177	0.169 ± 0.024	0.252 ± 0.057	0.782 ± 0.365	0.169 ± 0.062	0.207 ± 0.027
	IAN	0.093 ± 0.030 (100%)	tr	n.d.	tr	tr	n.d.
4-Methoxy-I3M-GSL		0.113 ± 0.025	0.060 ± 0.004	0.018 ± 0.004	0.049 ± 0.010	0.038 ± 0.016	0.038 ± 0.004
	4-Methoxy-IAN	0.044 ± 0.011 (100%)	n.d.	n.d.	n.d.	n.d.	n.d.
1-Methoxy-I3M-GSL		0.012 ± 0.009	0.243 ± 0.070	0.051 ± 0.022	0.012 ± 0.002	0.020 ± 0.011	0.009 ± 0.003
	1-Methoxy-IAN	0.001 ± 0.001 100%)	0.007 ± 0.002 (100%)	n.d.	n.d.	n.d.	n.d.

In brackets, the amounts of formed nitriles (%_CN_) concerning the initial GSL content are given.

Abbreviations: 3-but-GSL: 3-butenyl GSL; 2-OH-3-but-GSL: 2-hydroxy-3-butenyl GSL; 2-OH-3-but-CN: 3-hydroxy-4-pentenenitrile; 3-MTP-GSL: 3-(methylthio)propyl GSL; 4-MTB-GSL: 4-(methylthio)butyl GSL; 3-MSOP-GSL: 3-(methylsulfinyl)propyl GSL; 4-MSOB-GSL: 4-(methylsulfinyl)butyl GSL; tr: traces; n.d.: not detected.

**Table 2:** Calculated median growth inhibitory concentration of aromatic and methylthioalkylnitriles after 72 h treatment as determined in the WST-1 assay.

nitriles	IC_50_ [mM]
benzyl-CN	19.95
2-phenylethyl-CN	18.21
3-MTP-CN	19.49
4-MTB-CN	17.89
5-MTP-CN	12.19
6-MTH-CN	8.15
