# Supplementary material for: Cytotoxic and genotoxic potential of food-borne nitriles in a liver in vitro model
Source: Sci Rep. 2016 Nov 24;6:37631. doi: 10.1038/srep37631 (PMC5121622; doi:10.1038/srep37631)
Supplement: Supplementary Information [file srep37631-s1.pdf]

Ms.No.: SREP-16-15123

Cytotoxic and genotoxic potential of food-borne nitriles in a liver *in vitro* model

Franziska Kupke, Corinna Herz, Franziska S. Hanschen, Stefanie Platz, Grace A. Odongo, Simone Helmig, María M. Bartolomé Rodríguez, Monika Schreiner, Sascha Rohn, Evelyn Lamy

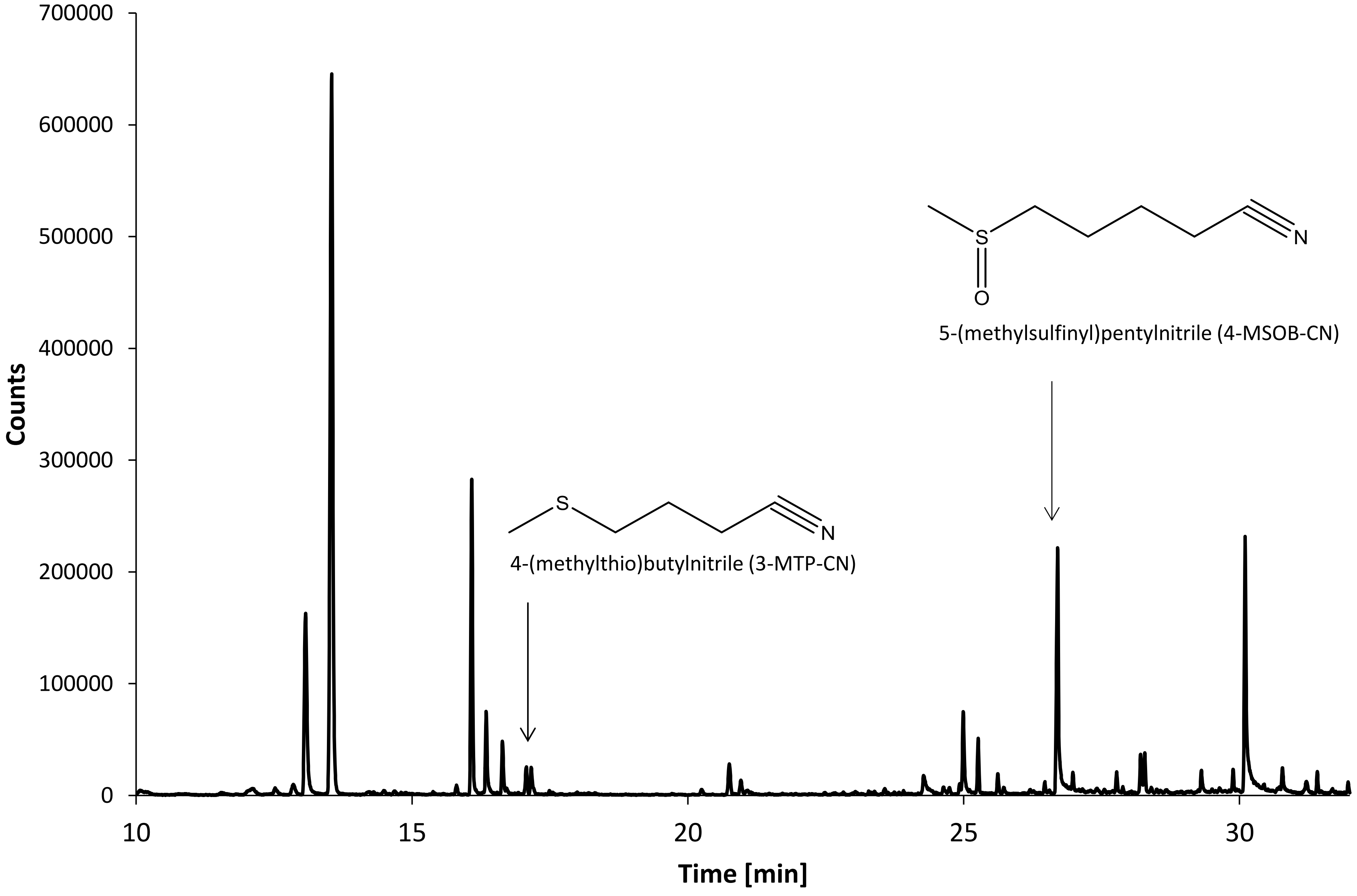

GC-chromatogramm of the breakdown products in broccoli; major nitriles were 4-(methylthio)butylnitrile and 5-(methylsulfinyl)pentylnitrile.

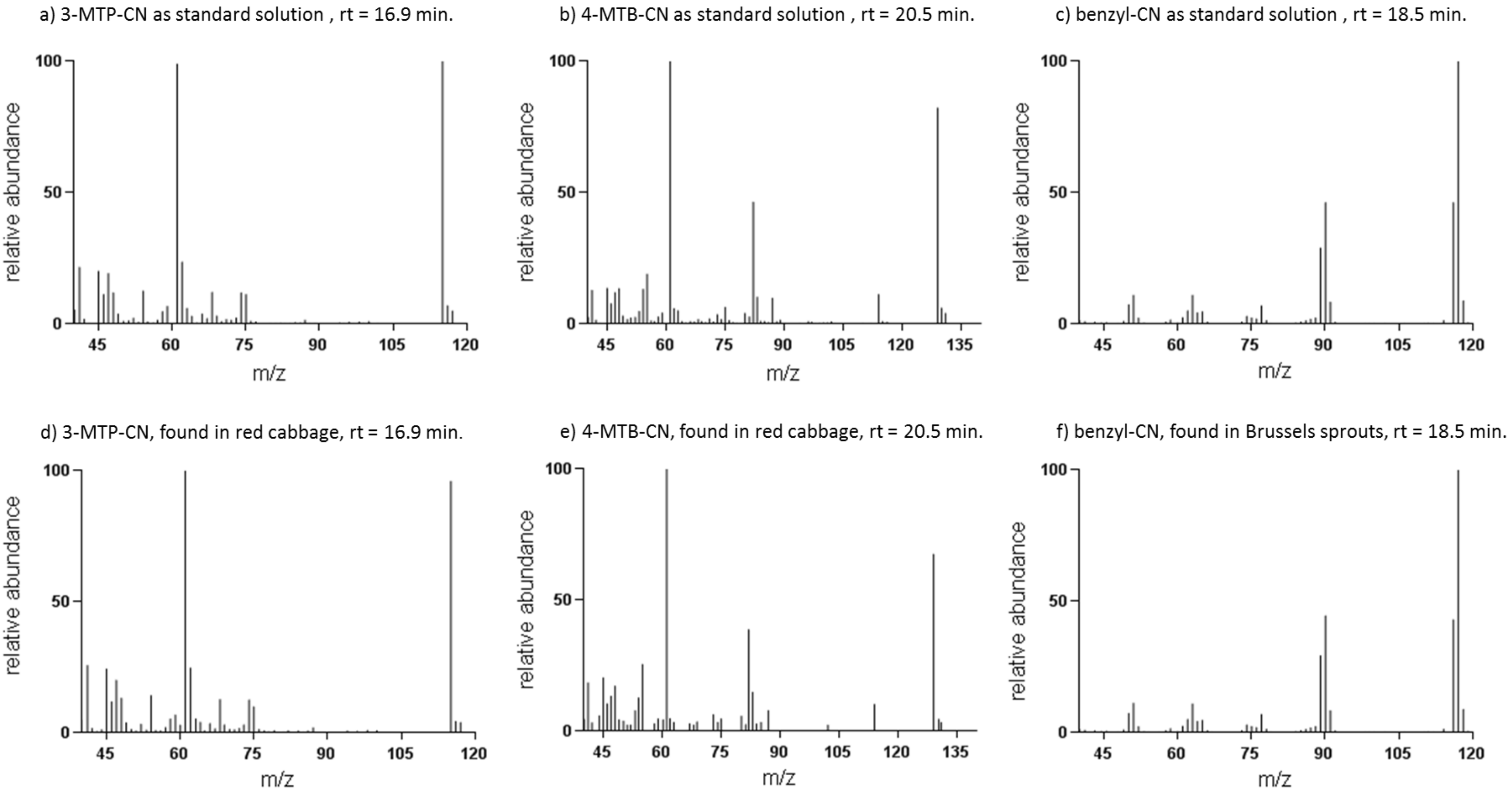

Mass spectra of 3-MTP-CN, 4-MTB-CN and benzyl-CN as standard solutions (a, b, c) and identified in red cabbage (d, e) respectively in Brussels sprouts (f).
